# Research Career Intentions Among Non-Native English-Speaking Graduate and Postdoctoral Trainees in STEM—Results from Cross-Sectional and Longitudinal Studies

**DOI:** 10.5539/ies.v15n3p61

**Published:** 2022-05-05

**Authors:** Hwa Young Lee, Shine Chang, Cheryl B. Anderson, Erin K. Dahlstrom, Carrie Cameron

**Affiliations:** 1 Cancer Prevention Research Training Program, The University of Texas MD Anderson Cancer Center, USA; 2 Department of Epidemiology, The University of Texas MD Anderson Cancer Center, USA; 3 Department of Behavioral Science, The University of Texas MD Anderson Cancer Center, USA

**Keywords:** non-native English, research career intention, mentoring, job-seeking behavior, STEM

## Abstract

International graduate trainees, many of whom are non-native English-speaking (L2) trainees, comprise more than half of graduate-level trainees in STEM, but little is known regarding factors that influence their career intentions, especially those that foster their growth as scientists to achieve their full potential in research. Thus, the purpose of our studies was to examine the relationship between L2 status and contextual factors that help shape the learning experiences and plans for research-focused careers. Study 1 collected cross-sectional survey data from doctoral and postdoctoral trainees (N=510) from research institutes in the Texas Medical Center in Houston. We examined which factors were associated with research career intentions using multiple linear regression analysis. Study 2 collected longitudinal data from doctoral and postdoctoral trainees (N=185) from 71 institutions in 33 states in the U.S. Repeated measures of career intentions were evaluated using mixed-effect modeling, and cross-tabulation analysis evaluated job-seeking behaviors by language status. Results showed that L2 trainees had stronger intentions to pursue research careers than did native English-speaking trainees (L1), controlling for other variables. Mentoring, trainee self-efficacy, and the perception of working more than mentors expected influenced each career intention differently. In Study 2, career intentions did not change over time for L2 or L1 trainees, but L2s preferred working in higher education or research institutes more than L1s. L2s, however, were more likely to be in early stages of seeking jobs compared to L1s. These findings provide implications for research mentors, advisors, and academic administrators in facilitating L2 career advancement and success.

## 1. Introduction

The number of international graduate students and postdoctoral fellows (hereafter, “trainees”, Note 1) in the U.S. has grown over the past decades, such that international trainees comprise about 20% of the graduate-level trainees in the U. S., with the majority of graduate trainees in STEM (Allum & Okahana, 2015). For example, 81% of graduate trainees in electrical and petroleum engineering programs and 79% in computer science programs were international trainees.

Non-native English (L2) speakers comprise a large proportion of international doctoral and postdoctoral trainees (Institute of International Education, 2019b). Most of the top 10 countries that send trainees to the U.S. are Asian countries, such as China, South Korea, Taiwan, and Vietnam (Institute of International Education, 2019a). While there is a great deal of research on the career intentions of domestic U.S. STEM trainees to persist in research careers (Fuhrmann et al., 2011; Gibbs et al., 2015), very little is known about how language status shapes the research career persistence and ambitions of L2 trainees (domestic or not). Previous studies have shown that, because language status is one of the most deep-rooted aspects of identity, it plays a greater role than hitherto recognized in the STEM career development (Cameron et al., 2020). Linguistic and socio-cultural competence are deeply intertwined, affecting virtually all aspects of academic life, from expression of opinions, challenging positions, and independent assertions, to attitudes about appropriate ways to approach and communicate with mentors and other authority figures, to participation in open discussion and customary organization of texts such as theses, manuscripts, and other documents (Angelova & Riazantseva, 1999). Viewed from the lens of Hofstede and Hofstede’s dimensions of national culture (Hofstede, 2011), many of these issues can be analyzed as expressions of Individualism (independence vs. interdependence), Power Distance (including Confucian values of duty to authority and family in the case of east Asians), and Hedonism (culturally determined attitudes about gratification of personal wishes). From this viewpoint, many national cultures from outside the U.S. can be described as having many commonalities and being distinct from U.S. culture. Complicated relationships among nationality, cultural differences, and language differences are a rich area for study, especially in relation to research career intentions; the current study focuses on understanding how L2 status (as a broad, discrete category) may be related to the successful transition from trainee to independent scientist.

### 1.1 Theoretical Framework: Social Cognitive Career Theory, Career Choice, and Career Behavior

Social cognitive career theory (SCCT; Lent et al., 1994) has been a widely used theoretical framework to better understand the career development process. In SCCT, self-efficacy beliefs play a central role in influencing interest, goals, and choices that are related to particular academic tracks and career fields that people pursue (Lent et al., 2018). Self-efficacy refers to beliefs about one’s ability to perform the behaviors necessary to attain particular goals, such as obtaining a graduate degree. Self-efficacy influences goal directed behavior by not only determining what goals to pursue, but also by deciding how much effort to invest in pursuing the goals and overcoming challenges, as well as to what extent to persist in the face of difficulties and obstacles (Bandura, 1997).

According to Lent, contextual or environmental factors that serve as proximal influences on critical choices influence self-efficacy (Lent et al., 1994). Contextual factors can function as barriers (e.g., discrimination, differential educational opportunities) or supports (e.g., supportive mentoring, opportunity structures) that inhibit or facilitate career goals and outcomes by directly influencing the active phases of choice-making (i.e., setting goals and translating them into action) and by indirectly influencing self-efficacy. In SCCT, person factors (e.g., language issues, gender, rank) are posited to affect *both* the primary and contextual influences on self-efficacy (Lent et al., 1994). Previous studies using SCCT, however, have not fully investigated research career intentions of graduate trainees in STEM, examining barriers and facilitators which influence graduate trainees differently based on language status. L2 language status could serve as an impediment to career progress by negatively affecting both the learning experiences that serve as the primary sources of self-efficacy and contextual factors that directly influence the goals and actions necessary for career advancement. On the other hand, L2s of cultural backgrounds that include high power distance and/or low hedonism—i.e., backgrounds which value duty to the mentor and to one’s parents as well as sacrifice of personal wants—could serve as a facilitator to behaviors that are necessary for career advancement. The mentoring received by L2 trainees could serve as both a contextual support and an important source of strengthening trainee self-efficacy. If the mentoring support is perceived to be high, then goal-behavior relations will be stronger, as evidenced by high research career commitment and intention by trainees (Lent et al., 1994). Thus, one of the purposes of the current paper was to examine how language status contributes to barriers trainees may face and how contextual support (e.g., mentoring) influences trainees’ research career intentions.

### 1.2 Language Status and Trainee Barriers

Besides political obstacles that can affect their immigration status and ability to complete their training, international L2 trainees encounter a variety of academic and social challenges that can be barriers to their graduate training and research career intentions (Chang & Kanno, 2010). These challenges, in part, are likely related to language issues, including a lack of English proficiency, different language styles (e.g., accents), and cultural differences related to language and communication (e.g., tendency to not interrupt a speaker, not speak up, not voice personal opinions). One study reported that the majority of L2 trainees took at least twice as much time to create a presentation, manuscript, abstract, or poster in English than they did in their native language, and many believed they were passed over for important presentations and even promotions because of their English proficiency or style (Cameron et al., 2011). L2 trainees from East Asian countries in particular have experienced marginalization or helplessness in class discussions more often than L1 trainees, although the two groups did not differ in their desire to participate in graduate-level classroom discussions (Yoon, 2013). Yoon’s study showed that L2 trainees had low confidence in speaking up and expressing their opinions in class discussions (e.g., worry about their accents and grammar). This could reflect lower English proficiency, but also still-developing intercultural competence, where what to say and when to say it, using proper tone, are necessary skills for successful social interactions. Developing intercultural competence, the cultural knowledge, skills, and attitudes that allow people to function effectively across cultures, takes time and can be a lifelong process (Deardorff, 2006).

### 1.3 Mentoring Support

Within STEM doctoral and postdoctoral training environments, mentors, who provide high quality career and psychosocial support for trainees, play a crucial role in helping trainees as they shape their research career intentions (Estrada et al., 2018; Fleming et al., 2013; Leggon, 2010). Recent literature claims that career mentoring carries more weight in traditional mentoring, and that psychosocial mentoring, including culturally responsive mentoring, is often overlooked (Mullen & Klimaitis, 2021; Byars-Winston & Dahlberg, 2020). Psychosocial mentoring entails mentors showing personal interest in trainee backgrounds and cultures, sharing their own academic challenges and failures, being sensitive to their trainees’ needs, and providing emotional support and boosts to trainee confidence (Mullen & Klimaitis, 2021). Understanding individual cultural values and cultural identity can be essential for psychosocial mentoring for any trainee population, but may be particularly important for L2 trainees (Chung et al., 2007).

Studies examining psychosocial mentoring for L2 trainees have revealed that international trainees (mostly L2) who perceived little respect from their mentors (e.g., that the mentor was unkind, dismissive, unavailable) were more likely to report higher levels of academic stress than did L1 trainees (Rice et al., 2009). Since many L2 trainees are from backgrounds where interdependence rather than independence is valued and/or rote learning rather than problem-based learning is the norm, the strong emphasis placed on individual initiative and problem-solving in the U.S. may also serve as a stressor to L2 trainees. These considerations suggest a potential role for psychosocial mentoring in developing trainee coping skills and resilience as it has been linked with reduced stress, a strong sense of belonging, and a more positive academic self-concept among trainees (Curtin et al., 2013; Wei et al., 2012). Additional studies have found that positive mentor-trainee relationships positively influence trainee career intention (Shen & Kram, 2011), career resilience (Arora & Rangnekar, 2015), and trainee positive self-concept, resulting in future career and job-seeking behaviors (Kao et al., 2020).

In addition to the nature of the mentoring, the quantity of attention from the mentor is important. Regular one-on-one meetings with a mentor may be critical. Questions asked about the availability of mentors are often included as items measuring psychosocial mentoring or career mentoring. Independent from the constructs of psychosocial or career mentoring, asking questions about one-on-one meetings with mentors allows more specific conclusions to be drawn. One-on-one meetings are likely important for trainees for several reasons: 1) trainees can receive specific and tailored feedback, 2) they can build a stronger relationship with their mentors by talking about research, other aspects of the doctoral program, and even personal issues, and 3) trainees and mentors have a better opportunity to align expectations when they meet regularly one-on-one; otherwise, they may not fully know or achieve each other’s expectations. Indeed, research has shown that one-on-one meetings with mentors enhance the relationship and make trainees more likely to complete their PhD degrees (Frasier, 2013; Heath, 2002).

### 1.4 Trainee Career Commitment Behaviors

Trainees contribute to successful mentoring relationships and must accept responsibility in these partnerships. Given the centrality of written and spoken products in advanced research settings, the trainee needs to be particularly receptive to their mentor’s guidance in scientific communication (SC; e.g., editing trainee’s manuscript, suggesting a writing class, presentation rehearsals). Mentors invest a considerable amount of time editing or writing comments when reviewing trainees’ manuscripts. If trainees do not follow their mentors’ guidance, the mentors may waste their time unnecessarily. On the other hand, some trainees may not receive enough guidance in some areas, such as in SC skills, even though they want it (Cameron et al., 2011). Thus, to improve mentoring support, trainees need to communicate with their mentors to confirm whether their mentor’s guidance aligns with the trainee’s expectations.

In addition to following the mentor’s guidance, trainees need to show effort toward their goal behaviors. Their effort behaviors could be attributed to intrinsic motivation to achieve their career goals or they may be an attempt to create a favorable impression. Mentors are in a powerful position to influence their trainees’ career development and introduce them to the world of academic research. Thus, trainees are likely to actively monitor their impression management techniques and behaviors to foster good mentor-trainee relationships. We argue that trainees with higher research career intentions would be more likely to create a favorable impression to their mentors, and that L2 trainees may be especially more likely to show effort to impress their mentors. Some qualitative studies have found that trainee efforts to impress mentors were associated with the pursuit of graduate degrees for international L2 doctoral trainees from Asian countries (Liu et al., 2015; Takashiro, 2017; Wang et al., 2016; Zhou, 2014).

### 1.5 Career Opportunities

L2 trainees (domestic or not) generally come to the U.S. mainland with clear career goals of continuing to pursue advanced training or employment (e.g., pursuing a postdoctoral fellowship, finding scientific research opportunities; (Hyun, 2019)). Although the National Science Foundation has reported how many immigrant workers entered the U.S. labor force based on issuing temporary work visas (National Science Board, 2019), only a few studies have been conducted specifically on the job-seeking behaviors of L2 graduate trainees in the U.S. For example, Coffey and colleagues indicated that L2 graduates have revealed significant barriers to finding employment, including their visa status, lack of work experience, and discrimination (Coffey et al., 2018). Since L2 graduate trainees make up more than half of the trainees in STEM graduate programs, more studies are needed among doctoral- and postdoctoral-level L2 trainees to better understand their job-seeking behaviors.

## 2. Research Problems and Research Questions

L2 research trainees are a vital part of the U.S. scientific research workforce and contribute invaluable knowledge and expertise to STEM research. It is critically important to understand factors that influence their career decisions and the quality of their training, especially those that foster their growth as scientists, to reduce attrition and achieve the full potential of the research enterprise. However, few studies, to our knowledge, have compared research career persistence intentions of L2 trainees to those of L1 trainees. As part of a larger, multi-year study investigating the role of communication skills in STEM research career development of several linguistic groups, including L1 speakers of Standard Academic English, L1 and bilingual speakers of non-standard Academic English, and L2s (Cameron et al., 2020), the current study focuses specifically on the impact of L2 status on these social-cognitive career factors. Using both cross-sectional (Study 1) and longitudinal (Study 2) survey data, we sought to elucidate the interplay of L2 status, mentoring factors related to trainee research career intentions and trainee self-efficacy, and trainee commitment behaviors. This work extends previous studies in the field examining associations between language status of trainees and academic achievement/satisfaction, most of which used qualitative data and had small samples. The findings provide insights for research mentors, advisors, and academic administrators on intentional strategies to support L2 trainee persistence and success.

For these reasons, we designed two studies to answer the following research questions.

1. Is language status related to research career intentions? (Study 1 & Study 2)
2. Are mentoring, trainee self-efficacy, and trainee commitment behaviors related to trainees’ research career intentions? If so, which types of career intentions? (Study 1)
3. Are there language status differences on how mentors guide their students on SC tasks and whether students follow this guidance? (Study 1)
4. Examining both career role and desired work setting, do research career intentions change over time, and are there language differences? (Study 2)
5. Do job-seeking behaviors differ by language status? (Study 2)

## 3. Study 1: Method

### 3.1 Participants and Procedure

Trainees from research institutes in the Texas Medical Center in Houston were invited to an online survey and 510 trainees met the eligibility criteria of being 18 years or older and doctoral or postdoctoral fellows with research as their primary training focus. Participants agreed to participate via online informed consent, and the Institutional Review Board of The University of Texas MD Anderson Cancer Center approved the study protocol (Protocol 2009-0409). They completed an online survey in 2012-2013 and received a $20 gift card as compensation for completing the survey, which took 20-30 minutes (Cameron et al., 2015). The sample characteristics are shown in Table 1.

### 3.2 Instrument

We developed an online survey to explore trainee research career intentions, trainee attitudes, and behaviors related to mentoring in SC reflecting previous related research (Lent et al., 1994; Longo et al., 2011; Ragins & McFarlin, 1990). The survey included questions about trainees’ career intentions, trainee report on their mentors’ mentoring functions (career and psychosocial), regular one-on one meeting with their mentors, trainee perception of working more than a mentor expects, trainee behaviors following a mentor’s guidance in SC, and demographic characteristics.

#### 3.2.1 Career Intentions (Outcomes)

Trainees’ intentions to pursue a research career were measured according to their intentions to 1) pursue the next-step position in research, e.g., a postdoctoral or junior faculty position; 2) conduct and lead their own research and research teams (PI intention); or 3) participate in conducting research as a staff member. These items were rated on a Likert-type scale ranging from 1 (strongly disagree) to 5 (strongly agree), with higher scores reflecting trainees’ stronger intentions.

#### 3.2.2 Self-Efficacy

A 22-item scale was developed to measure trainees’ confidence in their ability to complete scientific writing, presenting, and speaking tasks (Anderson et al., 2016). These items were measured with a 5-point Likert-type scale ranging from 1 (strongly disagree) to 5 (strongly agree). The coefficient alpha of the 22 items was .94 and the mean score averaging 22 items was highly associated with three sub-scale scores of writing, presenting and informal speaking (*r* = .77 to .90). Thus, a mean score averaging all 22 items was used to avoid multicollinearity issues that may arise when using three subscales of self-efficacy in a regression analysis. Example items of writing, presenting, and informal speaking are “Excel in scientific writing tasks, e.g., abstracts, manuscripts,” “Give an oral presentation at a scientific meeting,” and “Effectively answer questions from the audience at a scientific meeting.”

#### 3.2.3 Mentoring Variables

We examined three types of mentoring variables: two mentoring functions, and regular one-on-one meetings, measured as self-reports from trainees. The assessment of mentoring was developed by adapting items from Ragins and McFarlin’s mentor role instrument (Ragins & McFarlin, 1990) and our focus groups (Cameron et al., 2013). We conducted confirmatory factor analysis to determine the construct validity of an initial item pool of 18 items reflecting mentoring functions in two areas: career mentoring (7 items) and psychosocial mentoring (11 items). Results indicated that a two-factor model (career mentoring and psychosocial mentoring) had acceptable fit, χ^2^ S-B: (89)=232.37. *p*< .001, root mean squared error of approximation (RMSEA)= .06, 90% CI ( .05 - .07), comparative fix index (CFI)= .95, Tucker-Lewis Index (TLI)= .94, standardized root-mean-square residual (SRMR)= .04, after deleting three psychosocial items which were cross-loaded on career mentoring with content overlap across items (Hu & Bentler, 1999). Example items of career mentoring and psychosocial mentoring are “Introduces me to important people in the field,” and “Discusses my concerns regarding such issues as feelings of competence, commitment to advancement, relationships with peers and superiors, or work/family conflicts.” The coefficient alpha of the 7 career mentoring items was .89, and that of the 8 psychosocial mentoring items was .89, allowing us to make a scale score for each dimension (Cortina, 1993).

Regular one-on-one meeting was assessed with one self-reported item using a 5-point scale ranging from 1 (never) to 5 (always).

#### 3.2.4 Trainee Commitment Behaviors

Trainee commitment behaviors were measured by the perception of working more hours than a mentor expects and the degree to which trainees followed their mentor’s guidance about developing their SC skills. Trainee perception of working more than a mentor expects was assessed by asking if trainees had routinely worked more hours per week than their mentors had expected. Responses were rated with a 5-point Likert-type scale ranging from 1 (strongly disagree) to 5 (strongly agree).

#### 3.2.5 Mentor Guidance Behaviors by Trainees

For trainees’ behaviors following mentors’ guidance, 8 items were used to measure how trainees follow their mentors’ guidance on SC skills—writing, presenting, and informal speaking. Results of confirmatory factor analysis indicate that a one-factor model had acceptable fit, χ^2^ S-B (9)=46.67, *p*< .001, CFI= .95, TLI= .91, RMSEA= .10, 90% CI ( .07 - .12), SRMR= .04, after deleting two items due to content overlap. These items were measured with a 5-point Likert-type scale ranging from 1 (strongly disagree) to 5 (strongly agree). An example item is: “I seek out my mentor’s advice on my writing at all times and try to learn from their advice.” The coefficient alpha of these items was 0.85, and the composite score of the behaviors was used. Additionally, there was one separate response option of “my mentor doesn’t do this.” This allowed us to contrast mentors’ mentoring behaviors on SC tasks between L1 and L2 trainees. Frequencies were collected on this option to determine L1 and L2 differences.

### 3.3 Research Design

A correlation analysis was performed to examine whether trainee demographic variables (language status, gender, and rank) and career intention variables were associated with self-efficacy, mentoring support, and trainee commitment behaviors. For the analysis, gender was coded as “0” if the trainee was female and “1” if the trainee was male. Language status was coded as “0” if the trainee was L1 and “1” if the trainee was L2. Academic rank was coded as “0” for doctoral trainees and “1” for postdoctoral fellows. Significant positive values indicated that the focal group was highly correlated with the variables of interest.

A multiple linear regression was performed for each career intention using IBM SPSS Statistics Software, v22 (Armonk, New York). Although career intention variables are somewhat related to each other, the purpose of the analysis was to identify which variables were significant predictors of each intention. We included demographic variables (language status, gender, and rank), self-efficacy in SC tasks, mentoring variables (career mentoring, psychosocial mentoring, one-on-one meeting), and trainee commitment behaviors variable in each career intention model.

Analysis of variance (ANOVA) was conducted to examine whether trainees’ following their mentors’ guidance on SC tasks differed by language status, and whether their mentors’ guidance on SC was different between L1 trainees and L2 trainees.

## 4. Study 1: Results

### 4.1 Multiple Linear Regression

As shown in Table 2, the correlation analysis showed that in general, all variables of interest were significantly related to at least one of three research career intention variables and significant associations varied by each research career intention. Compared to L1 trainees, L2s had lower self-efficacy in SC, perceived that they worked more than a mentor expected, and had stronger research career intentions.

Table 3 shows results of multiple regression analyses for the three research career intentions. Regarding the intention to pursue the next-step position, providing more psychosocial mentoring (*b*= .30, *p* < .01), frequent one-on-one meetings (*b*= .13, *p* < .01) were significantly associated with higher intention to pursue the next-step position. The R^2^ indicates that 7% of the variation in the intention to pursue the next-step position can be explained by the model.

Regarding the intention to conduct and lead research and a research team (PI intention), L2 trainees rather than L1 trainees (*b*= .40, *p* < .01), receiving more psychosocial mentoring (*b*= .41, p < .001) and more frequent one-on-one meetings (*b*= .11, *p*= .04), and having greater perception of working more than a mentor expects (*b*= .16, *p* < .01) had significantly higher intention to be a PI. The R^2^ indicates that 14% of the variation in the intention to be a PI can be explained by the model.

Regarding the intention to conduct research as a staff member, language status and academic rank were significant predictors. L2 trainees rather than L1 trainees (*b*= .25, *p* =.04) and doctoral trainees (*b*= −.32, *p* < .01) were significantly related to this intention. Self-efficacy was negatively related to this intention (*b*= −.34, *p* < .001); trainees who had lower self-efficacy in SC were more likely to indicate intention to conduct research as a staff member. This model explained 8% of the variation in the intention to conduct research as a staff member.

Our results indicate that controlling for other variables, L2 trainees had significantly stronger research career intentions than L1 trainees. That L2 status was significantly and positively correlated with working more than their mentor expected further emphasizes the commitment of the L2 trainees for both types of research careers. Similarly, L2 status was significantly and negatively correlated with self-efficacy in SC. This may imply that although L2s had lower self-efficacy in their English writing and speaking skills than L1s, they saw a career in research as a staff member, rather than the research leader, as a viable and desirable option which allowed them to stay in a research-oriented career. Psychosocial mentoring was important for both intentions to pursue the next-step position (i.e., short-term goal) and PI intention (i.e., long-term goal), and there was no relation to language, indicating that mentoring was important for both L2 and L1 trainees.

### 4.2 ANOVA

As shown in Table 4, L2 trainees (M_L2_=4.12) followed their mentor’s guidance significantly more than L1 trainees (M_L1_=3.87; F=15.47, p<.001). At the same time, mentors mentored their L1 trainees (M_L1_ =3.02) far less often than they did their L2 trainees (M_L1_ =1.00). The two things that mentors did not do or ask of their L1 trainees most frequently were to suggest that they take classes in writing and that they rehearse their pronunciation.

## 5. Study 2: Method

### 5.1 Participants and Procedure

Participants were doctoral and postdoctoral trainees in biomedical and behavioral sciences from 71 institutions in 33 states in the U.S. Eligibility criteria for trainees were the same as for Study 1, but for Study 2, we first invited mentors who were currently mentoring doctoral or postdoctoral trainees through e-mails. Once mentors agreed to participate in the study, they were asked to nominate their trainees who were eligible for our criteria. Our study team contacted trainees via e-mail to ask for their participation in the study. One hundred eighty-five trainees met our eligibility criteria and completed online informed consent and surveys. The trainee participants were asked to complete four online surveys during August 2014 to November 2016. The attrition rate over time was 16%. The study was approved by The University of Texas MD Anderson Cancer Center’s Institutional Review Board (Protocol 2013–0829). The sample characteristics are shown in Table 5.

### 5.2 Online Instrument

#### 5.2.1 Career Intentions

Two aspects of trainees’ career intentions (desired career role and desired work setting) were assessed repeatedly at four time points over the 2-year study period. The time interval between each survey was 7-8 months. Career roles were measured using three items: “For my role in research, I intend to 1) conduct and lead my own research and research teams (PI intention), 2) participate in conducting research as a staff member, or 3) support science and research (e.g., be a teacher, science administrator, grants manager, science policy analyst, science reporter).” Work settings were measured using two items: “As of today, I would like to work in 1) a higher education academic setting (e.g., university, graduate school, medical school, school of public health, other health professional school) or 2) a research institute (e.g., Cold Spring Harbor Laboratory, Broad Institute, Pennington Biomedical Research Center).” Career intention items were rated on a Likert-type scale ranging from 1 (strongly disagree) to 5 (strongly agree), with higher scores reflecting trainees’ stronger intentions.

#### 5.2.2 Job-Seeking Behaviors

To obtain additional information about research career intentions between L1 and L2 trainees, we asked one question about job-seeking at the fourth timepoint: “Regarding your most desired career path, which of the following describes your current situation?” Possible responses were: 1) no current job-application behavior because it is too early; 2) no current job application, and I have not started looking for a job yet; 3) searching for positions; 4) submitting applications; 5) doing job interviews; 6) deciding on one or more job offers; and 7) accepted a position.

### 5.3 Research Design

The five research career intentions were assessed using mixed-effects linear models with the intercept as a random effect. The models were estimated using maximum likelihood via PROC MIXED in SAS (v 8.2). For each intention outcome, three subsequent models were estimated and compared to identify a best-fitting model. The first model for each intention included only the time variable to examine whether career intentions changed over time. The second model, which added time as a random effect, was used to estimate whether the average changes in a career intention vary across trainees. The third model added language status to estimate if trainee language status is related to research career intentions. To determine which of the nested models was a good-fitting model, several fit indices were compared: Akaike’s information criterion (Akaike, 1987), Akaike’s information criterion for small samples (Bozdogan, 1987), and the Bayesian information criterion (Schwarz, 1978). Smaller values for these indices suggest a good-fitting model.

For job-seeking behavior outcomes, we used a cross-tabulation analysis to reveal differences in job-seeking behaviors at Time 4 by language status, after controlling for trainee rank. Since trainees who have been in graduate programs longer than those who just started are more likely to be searching for a job, the number of years they have been in their graduate program should be considered. Although we assessed only the number of years a trainee had worked with their mentors for the study, this variable is considered highly correlated with the number of years in their graduate programs. Thus, we first examined whether the number of years trainees had worked with their mentors differed by language status.

## 6. Study 2: Results

### 6.1 Mixed-Effects Models

Three nested models for each career intention were compared using fit indices and model interpretation. Table 6 shows only the final model for each intention. The final model for the career intention to conduct and lead research and a research team (PI intention) was the model with trainee language status and time as fixed effects, and time as a random effect; here, PI intention, on average, did not change over time nor differ by language status (fixed effect_time_ = −.05, *p* = .10; fixed effect_language status_= .30, *p*=.07), but rates of change in the PI intention (i.e., random slope) were different across individual trainees (random slope = .02, *p*= .01). On the other hand, intention to support science and research decreased over time (fixed effect = −.08, *p*= .03), rates of change in this intention also varied across individual trainees (random slope = .04, *p*= .01), but language status did not predict the intention to support science significantly. Neither time nor language status significantly predicted intention to conduct research as a staff member, indicating no change over time on this career intention and no differences by language status.

Trainees’ preferred work settings did not change over time (fixed effect_research institute_ = −.04, *p*= .32; fixed effect_higher edu_= −.05, *p*= .25). However, on average, L2 trainees preferred working in a research institute more than L1 trainees (fixed effect = .59, *p*<.*001*) and a higher education academic setting (fixed effect = .39, *p*= .02).

### 6.2 Trainees’ Job-Seeking Behaviors

Overall, L2 trainees tended to be in earlier stages of job seeking compared to their L1 peers. A significance level was not provided because the sample sizes for particular categories were small. As shown in Table 7, among doctoral trainees, 17 (81%) of the L2 trainees (n = 21) versus 54 (62%) of the L1 trainees (n = 87) were in the early stages of seeking jobs. Only one L2 doctoral trainee versus six L1 doctoral trainees was deciding on one or more job offers or had accepted a position (i.e., late stages). This pattern was similar between postdoctoral L1 and L2 trainees: 9 of 20 L2 postdocs (45%) were still in the early stages of seeking jobs, whereas only 3 of 22 L1 postdocs (14%) were. Further, only 3 of 20 L2 postdocs (15%) were deciding on one or more job offers or had accepted a position (late stages), but a higher proportion of L1 postdocs (7 of 22, 32%) were at these stages. There were no differences between L1 and L2 doctoral and postdoctoral trainees in terms of disciplines and the number of years that trainees had worked with their mentors.

In sum, Study 2 results indicate that career intentions did not change over time for L2 or L1 trainees, but L2s preferred working in higher education or research institutes more than L1s. L2s, however, were more likely to be in early stages of seeking jobs compared to L1s after accounting for time in rank.

## 7. Discussion

Our Study 1 extended previous research on social cognitive career theory by examining whether the scope of application of this theory can be applied to more diverse populations, such as L2 trainees, and highlights the roles of the contextual environment and culture in shaping career development (Brown & Lent, 2017). In addition, our Study 2 examined whether L2 trainees’ career intentions compared to L1’s changed over time using multiple types of career intentions, and whether job-seeking behaviors differed by language status.

### 7.1 Career Intentions Between L1 and L2 Trainees

In Study 1, we found that L2 trainees had significantly stronger research career intentions than L1 trainees, regardless of whether they intended to lead their own research or conduct research as a staff member. Most participants from Study 1 were based in the Texas Medical Center in Houston, Texas, at research institutes. Similar to our findings in Study 1, we observed that L2 trainees in Study 2 from a national sample also had strong intentions to work at a research institute and in a higher education setting. These results imply that L2 trainees in biomedical doctoral and postdoctoral programs have strong desires to become researchers or scientists whether or not they lead research, and that such intentions remain high throughout training.

Gibbs and Griffin (2013) found that PhD trainees’ interests in academic careers (e.g., faculty positions) decreased as their training progressed. In Study 2, we did not observe a decline over time in both of the two aspects of research career intentions examined. Only trainee career intention to support science and research (e.g., be a teacher, science administrator, grants manager), which was inversely related to intention to be a PI, decreased over time. Interestingly, looking at only the PI intention of L1 doctoral and postdoctoral trainees, intention to be a PI seemed to decrease over time (data not shown) consistent with the results of Gibbs and Griffin (2013). After including both L1 and L2 trainees in our mixed-effects model, however, intention to be a PI did not decrease over time but rates of change in trainees’ PI intentions varied among trainees. The precursors and longitudinal correlates of this variability remain to be explored.

### 7.2 Mentoring Effects on Trainees’ Career Intentions

Our results showed that mentoring is an integral factor in trainees’ decisions to pursue a research career. Mentors’ psychosocial mentoring, in particular, was a significant predictor of trainee research career intention, regardless of whether intention was short-term (next-step position) or long-term (PI intention). Psychosocial mentoring encompasses the interpersonal aspects of the mentoring relationship that enhance a trainee’s sense of competence, as well as their effectiveness and identity in professional roles (Kram, 1985). In research training, the role of psychosocial mentoring in motivating trainees and sustaining their persistence in research careers may be especially important. Notably for L2 trainees, who may be subject to microaggressions and identity threats to their professional goals, psychosocial mentoring may serve as a crucial source of learning (Bandura, 1997) providing vicarious learning and social persuasion to build the self-efficacy needed for a research career.

Frequent one-on-one meetings with mentors played a more pivotal role in helping trainees achieve short-term career goals (i.e., next-step position), rather than long-term goals. It makes sense that in the intense training that takes place during the relatively brief duration of a doctoral program or postdoctoral fellowship, the primary goals for both mentor and trainee are to complete the requirements for these programs and to prepare for and secure the next career step. Frequent one-on-one meetings should facilitate this process and allow these goals to be met.

### 7.3 Trainee Commitment Behaviors

Trainee perceptions of working more than a mentor expects was a significant predictor of intention to conduct and lead research/a research team (PI intention). It was significantly different from total hours spent working per week, which was not related to any of the research career intentions. Thus, trainee perceptions of mentors’ recognition and acknowledgment of trainees’ working hours (or efforts) appears to be critical to the pursuit of their desired career paths, and this was significantly associated with L2 status. This could also reflect trainees’ intrinsic motivation to work hard to pursue an independent research career. Both goal setting theory (Locke & Latham, 1990) and SCCT (Lent et al., 1994) acknowledge that one’s goal commitment level, that is one’s determination to reach a goal, moderates the goal-action relation. When goal commitment is high and the goal is specific, people perform more goal-related behaviors.

Additionally, theory and years of research on self-presentation have shown that individual differences in people’s concern with public image and social evaluation affect their behavior when the importance of others’ evaluations is salient (Leary, 1995; Leary et al., 2011). Cultural psychologists have noted clear differences in self-construal among people from individualistic (e.g., independent self) and collectivistic (e.g., interdependent self) cultures (Markus & Kitayama, 1991). Likewise, culture determines how the self is presented. Previous research has shown that the self-enhancement bias (tendency to promote a positive view of the self) is present in Asian culture as well as in Western (Brown & Kobayashi, 2002). The key issues seem to be what is socially valued and how to show it. Brown and Kobayashi (2002) maintain that ‘culture mandates what is good and important’ and that ‘if culture values diligence, then people will claim to be industrious.’ Our data, where the L2 group was largely Asian in Study 1, supports this contention and provides new evidence for cultural differences in self-presentation behaviors.

### 7.4 Trainees’ Job-Seeking Behaviors

Our results regarding trainees’ job-seeking behaviors showed that L2 trainees, regardless of rank, spent more time than L1 trainees contemplating starting their job searches. Moreover, more L1 trainees than L2 were deciding on one or more job offers or had accepted a position. Although the number of L2 trainees who completed this question in our survey was not enough to generalize, the results could mean that L2 trainees are not confident enough to apply for a position or that they think that they are not ready even though their intentions to pursue a research career were relatively strong. In addition, for L2 trainees, not only language but also cultural barriers could influence their career decisions. Recent studies have shown that cultural differences in job applicants have an impact on employment opportunities in science and higher education (Bencharit et al., 2019; White-Lewis, 2020).

## 8. Conclusions

The findings of this study demonstrate that L2 graduate and postdoctoral trainees cannot be assumed to hold similar career intentions, beliefs, and behaviors as those of L1 trainees, and that different approaches to mentoring this population should be considered. Although there have been important advances in recognizing the need for strategies to increase gender, racial, and ethnic diversity in graduate education and STEM fields in the U.S., graduate training programs may assume a certain homogeneity among trainees in their needs, attitudes, and goals. Almost completely lacking in the literature and in practice is examination of L2 trainees’ needs, attitudes, and goals, which differ along linguistic, sociolinguistic, and sociocultural dimensions and represent rich opportunities for positive intervention. What few studies exist are often descriptive or do not have sufficient sample sizes for extensive analysis. The use of two types of samples here (cross-sectional and longitudinal) allowed us to integrate results and conclude that L2 trainees’ career intentions are different from those of L1 trainees, that factors affecting career intentions depend on the type of career intention, and that trainee career intentions do not seem to diminish over time in the way that L1 trainees’ intentions do. More importantly and positively, mentors can be culturally responsive to L2 trainees’ needs and facilitate L2 trainees’ motivation, research career interest, and job-seeking behaviors. To attain the goal of broadening and diversifying the STEM research workforce, recognizing the unique aspects of L2 trainees’ behaviors, attitudes, and goals will be a key advance.

## 9. Limitations and Future Directions

The present studies had some limitations. International L2 trainees face significant challenges from immigration policies and regulations that are not relevant for domestic L2 trainees. Immigration policies can influence international L2 trainees to choose career paths for which work visas are easily available after graduation, even if such paths are not of intellectual interest or preference. Data for visa status, intention to remain in the U.S. long-term, or how legal status influences career intentions were not available for this study. Similarly, in Study 2, a balanced sample for certain demographic characteristics (e.g., language status) was unavailable, limiting addition of more predictors of career intentions to the model and examination of associations between each job-seeking category and these characteristics. This can be addressed in future studies.

We are cognizant of cultural differences between L1 and L2 trainees and among L2 trainees. Although this study did not directly examine national cultural differences, we have relied on L2 status as an imprecise proxy for national culture(s) and have found significant differences between L2 as a group and L1 as a group. More specialized study of national differences is likely to reveal additional important information. In addition, we did not ask L2 trainees whether they intended to stay in the U.S. or to return to their home country after graduation. Thus, it is not clear whether they considered returning to their country to pursue a research career when they answered the question about job-seeking behaviors.

Given our finding that language status is related to trainee career intentions and job-seeking behaviors, further research is needed to explore other possible correlates and mechanisms of influence. Identifying other relevant and actionable variables would help mentors work more effectively with individual trainees, especially L2s. In addition, research to explore why there might be a discrepancy between career intentions and job-seeking behaviors, and why job-seeking behaviors differ by language status is needed. Together with the findings of this study, such research can help us find ways to work towards fully integrating these individuals into the research workforce and benefit from their contributions.

## Figures and Tables

**Table 1.** Study 1 sample characteristics

Variable	Category		Frequency	Percentage
Citizenship	USA		280	55
	Visa holder		230	45
Gender	Female		316	62
	Male		194	38
Rank	Doctoral		257	50
	Postdoctoral		233	46
Country raised	China		81	16
	USA (Puerto Rico)		214 (9)	44
	India		60	12
	Countries of Europe		34	7
	Countries of Asia (except for China)		56	11
	More than two countries		4	.8
	Other countries		48	10
	Missing		4	.8
Discipline	STEM		482	95
	Other (not specified)		28	5
Language status	English		255	50
	Others	Chinese (e.g., Cantonese, Mandarin)	81	32
		Spanish	25	10
		Hindi, Bangla, Marathi	28	11
		Other languages	55	22
		Missing	33	13

**Table 2.** Correlations among mentoring variables, trainee variables, career intentions, and demographic characteristics (N = 510 for Study 1)

	M (SD)	1	2	3	4	5	6	7	8	9	10	11
1	-	1										
2	-	0.15**	1									
3	-	0.16**	0.01	1								
4	3.68 (0.63)	−0.28**	0.06	0.03	1							
5	3.68 (0.67)	0.08	0.08	0.01	0.17**	1						
6	3.85 (0.69)	−0.03	0.06	−0.08	0.28**	0.45**	1					
7	3.59 (1.09)	0.07	0.02	−0.02	0.10*	0.25**	0.18**	1				
8	3.23 (0.94)	0.17**	0.10*	−0.08	0.03	0.10*	0.15**	0.16**	1			
9	4.16 (1.08)	0.08	0.08	−0.01	0.02	0.09	0.20**	0.16**	0.12*	1		
10	3.70 (1.27)	0.18**	0.14**	0.08	0.08	0.10*	0.24**	0.16**	0.19**	0.61**	1	
11	2.88 (1.16)	0.14**	−0.03	−0.13**	−0.19**	0.03	0.04	0.06	0.02	0.03	−0.08	1

*Note*. * *p* < .05; ** *p*< .01. 1= Language (0= L1); 2= Gender (0=female); 3= Rank (0=doctoral); 4= Self-efficacy; 5=Career mentoring; 6=Psychosocial mentoring; 7= Frequency of one-on -one meetings scheduled in advance; 8= Worked more than mentor expected; 9= Intention to pursue the next-step position; 10= Intention to conduct and lead research/team; 11= Intention to conduct research as a staff member.

**Table 3.** Summary of a series of multiple regression analyses for variables predicting research career intentions (N = 510 for Study 1)

	Intention to pursue the next-step position			Intention to conduct and lead research/team			Intention to conduct research as a staff member		
	b (se)	B	t	b (se)	Beta	t	b (se)	Beta	T
Language	0.11 (0.11)	0.05	0.95	0.40** (0.13)	0.16	3.22	0.25* (0.12)	0.11	2.12
Gender	0.12 (0.11)	0.05	1.13	0.22 (0.12)	0.08	1.83	−0.10 (0.11)	−0.04	−0.90
Rank	0.01 (0.10)	0.01	0.13	0.21 (0.12)	0.08	1.83	−0.32** (0.11)	−0.14	−2.93
Self-efficacy	−0.05 (0.09)	−0.03	−0.54	0.12 (0.10)	0.06	1.17	−0.34*** (0.09)	−0.18	−3.62
Career mentoring	−0.05 (0.09)	−0.03	−0.57	−0.11 (0.10)	−0.06	−1.16	0.03 (0.09)	0.02	0.31
Psychosocial mentoring	0.30** (0.09)	0.19	3.49	0.41*** (0.10)	0.22	4.27	0.13 (0.09)	0.07	1.37
One-on-one meetings	0.13** (0.05)	0.13	2.66	0.11* (0.05)	0.10	2.08	0.05 (0.05)	0.05	1.04
Worked more than mentor expected	0.06 (0.06)	0.03	1.14	0.16** (0.06)	0.12	2.63	−0.03 (0.06)	−0.02	−0.42
R^2^	0.07			0.14			0.08		
F	**4.03** ***			**8.58** ***			**4.54** ***		

*Note*. * *p*< .05, ** *p*< .01, *** *p*< .001. A *p* value of a variable was considered statistically significant.

Abbreviations: L1 = native English-speaking; L2 = non-native English-speaking.

**Table 4.** ANOVA for following mentor’s guidance suggestions between L1 and L2 trainees

Variable	Mean (SD)		F	*p* value
	L1 trainee	L2 trainee		
Follows mentor’s guidance	3.87 (0.70)	4.12 (0.62)	15.47	<.001
Mentor doesn’t do or ask this of trainee (frequency)	3.02 (2.16)	1.00 (2.02)	105.59	<.001

**Table 5.** Study 2 sample characteristics

Variable	Category		Frequency	Percentage
Citizenship	USA		141	76
	Visa holder		44	24
Gender	Female		112	60
	Male		73	40
Rank	Doctoral		131	71
	Postdoctoral		54	29
Discipline	STEM		163	88
	Other		20	11
Language status	English		139	76
	Others	Chinese (e.g., Cantonese, Mandarin)	17	37
		Spanish	6	13
		Hindi, Bangla, Marathi	3	7
		Korean	4	9
		Others	16	35

**Table 6.** The final mixed-effects model for each career intention (N=185 for Study 2)

			Estimated coefficient (se)	t value (Z value for random effect)
Intention to conduct and lead research/team	Fixed effect	Intercept	3.85 (0.11)	36.68
		Time	−0.05 (0.03)	−1.68
		Language	0.30 (0.17)	1.80+
	Random effect	Intercept	0.81 (0.12)	6.99
		Time	**0.02 (0.01)**	2.28*
		Residual	0.46 (0.03)	12.56***
Intention to conduct research as a staff member	Fixed effect	Intercept	3.36 (0.11)	29.79***
		Time	0.02 (0.03)	0.62
		Language	0.15 (0.16)	0.94
	Random effect	Intercept	0.76 (0.10)	7.36***
		Residual	0.71 (0.05)	15.19***
Intention to support science	Fixed effect	Intercept	3.32 (0.12)	28.54***
		Time	**−0.08 (0.04)**	−2.22*
		Language	−0.14 (0.17)	−0.80
	Random effect	Intercept	0.65 (0.12)	5.20**
		Time	**0.04 (0.01)**	2.31*
		Residual	0.84 (0.06)	14.06***
Work in a research institute	Fixed effect	Intercept	3.77 (0.15)	24.49
		Time	−0.04 (0.04)	−0.99
		Language	**0.59 (0.16)**	3.62***
	Random effect	Intercept	0.67 (0.10)	6.84***
		Residual	0.54 (0.04)	12.19***
Work in a higher education setting	Fixed effect	Intercept	4.13 (0.15)	27.56
		Time	−0.05 (0.04)	−1.16
		Language	**0.39 (0.17)**	2.27*
	Random effect	Intercept	0.77 (0.11)	7.34***
		Residual	0.47 (0.04)	12.24***

*Note.* + .05<p<.08, * *p*< .05, ** *p*< .01 *** p<.001.

**Table 7.** Trainee job-seeking behaviors at Time 4 across language status, gender, and rank

		Job-seeking behavior							
		No current job application behavior		Searching for positions	Submitting applications	Doing job interviews	Deciding on one or more job offers	Accepted a position	Total
		Too early	Have not started looking yet						
Doctoral	L1-Female	24	11	16	3	0	0	2	56
	L1-Male	9	10	3	3	2	2	2	31
	L2-Female	6	8	1	1	0	0	1	17
	L2-Male	2	1	0	0	1	0	0	4
Postdoctoral	L1-Female	0	2	5	2	1	1	2	13
	L1-Male	1	0	2	1	1	0	4	9
	L2-Female	0	2	2	0	0	0	1	5
	L2-Male	3	4	3	1	2	0	2	15
Total	L1	34 (31%)	23 (21%)	26 (24%)	9 (8%)	4 (4%)	3 (3%)	10 (9%)	109 (100%)
	L2	11 (27%)	15 (37%)	6 (15%)	2 (5%)	3 (7%)	0	4 (10%)	41 (100%)

## Footnotes

Note 1. In STEM research in the United States, ‘trainee’ refers to a postgraduate trainee or a postdoctoral fellow who is working in an apprentice-like relationship to the independent investigator of the research team. ‘Trainee’ is used when referring to status as an academic candidate. STEM trainees are frequently concurrently working in the research team, where they are referred to, along with postdoctoral fellows, as ‘trainees’. We follow this custom in this research report.
